# 588 Utilizing a Quality Improvement Approach to Increase Compliance with Patient Positioning in the Burn Unit

**DOI:** 10.1093/jbcr/irac012.216

**Published:** 2022-03-23

**Authors:** Catherine E Gallagher, Julia C Slater, Elizabeth L Dale

**Affiliations:** University of Cincinnati Medical Center, Cincinnati, Ohio; University of Cincinnati Medical Center, Cincinnati, Ohio; University of Cincinnati Medical Center, Cincinnati, Ohio

## Abstract

**Introduction:**

Patient positioning, notably positioning patients in “anti-deformity positioning”, is a standard practice in burn rehabilitation as it assists with edema management, scar contracture prevention, and wound healing. Successfully and consistently providing proper positioning of burn patients requires the combined effort of the multi-disciplinary burn team. The primary goal at our center was to increase the frequency that patients were correctly positioned to over 90% at the time of random audits.

**Methods:**

At a medium-sized, academic burn unit, once to twice weekly audits were conducted by burn lead therapists on the compliance of proper patient positioning over a 6-month period. Using this data as a trigger, a quality improvement project was designed using the PDSA (Plan-Do-Study-Act) cycle to help identify reasons behind lack of compliance. Surveys were distributed to the therapy and nursing staff to identify any barriers to care. Effects on positioning compliance post-intervention were monitored.

**Results:**

In the 6 months prior to intervention, our patients were positioned correctly an average of 76% of the time. Therapy and nursing surveys identified the following barriers to care: Nursing needed more education on positioning, and the approach was too heavily reliant on nursing efforts alone. To address these barriers, therapists provided education to both day and night shift nurses, communicated daily about positioning expectations, shifted the project from a nursing approach to a multidisciplinary approach, and made changes in therapy workflow. Immediately following the intervention, the compliance rates were 91% for the first month and 85% for the second month.

**Conclusions:**

Coordinating efforts of the entire burn team improves consistency for positioning in burn patients. Utilizing the PDSA cycle allowed us to identify areas for improvement and to develop appropriate interventions aimed at both increased education for nursing staff and workflow improvements for our therapists. Following the completion of our interventions we were able to obtain an immediate improvement in our compliance with proper positioning of burn patients.

